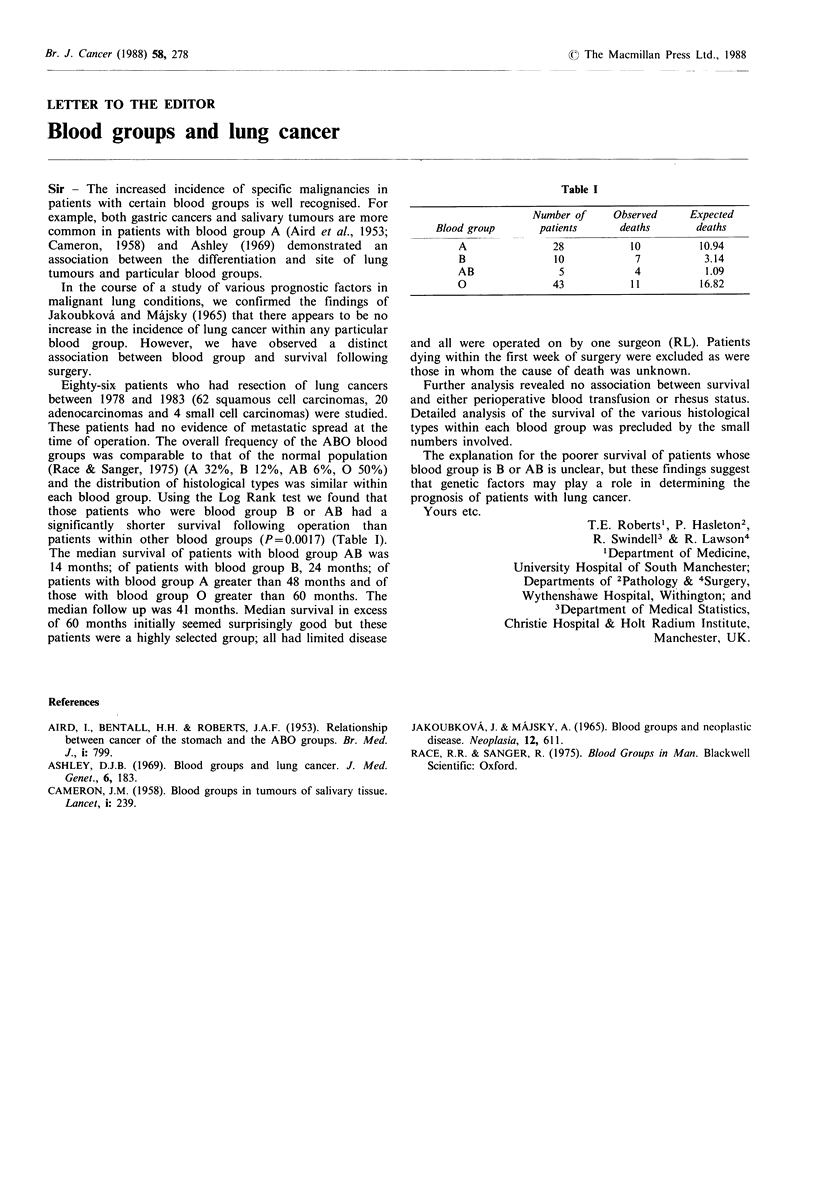# Blood groups and lung cancer.

**DOI:** 10.1038/bjc.1988.201

**Published:** 1988-08

**Authors:** T. E. Roberts, P. Hasleton, R. Swindell, R. Lawson


*Br. J. Cancer* (1988) **58**, 278 © The Macmillan Press Ltd., 1988

### LETTER TO THE EDITOR

### Blood groups and lung cancer

**Sir** – The increased incidence of specific malignancies in patients with certain blood groups is well recognised. For example, both gastric cancers and salivary tumours are more common in patients with blood group A (Aird *et al.*, 1953; Cameron, 1958) and Ashley (1969) demonstrated an association between the differentiation and site of lung tumours and particular blood groups.

In the course of a study of various prognostic factors in malignant lung conditions, we confirmed the findings of Jakoubková and Májsky (1965) that there appears to be no increase in the incidence of lung cancer within any particular blood group. However, we have observed a distinct association between blood group and survival following surgery.

Eighty-six patients who had resection of lung cancers between 1978 and 1983 (62 squamous cell carcinomas, 20 adenocarcinomas and 4 small cell carcinomas) were studied. These patients had no evidence of metastatic spread at the time of operation. The overall frequency of the ABO blood groups was comparable to that of the normal population (Race & Sanger, 1975) (A 32%, B 12%, AB 6%, O 50%) and the distribution of histological types was similar within each blood group. Using the Log Rank test we found that those patients who were blood group B or AB had a significantly shorter survival following operation than patients within other blood groups ($P=0.0017$) (Table I). The median survival of patients with blood group AB was 14 months; of patients with blood group B, 24 months; of patients with blood group A greater than 48 months and of those with blood group O greater than 60 months. The median follow up was 41 months. Median survival in excess of 60 months initially seemed surprisingly good but these patients were a highly selected group; all had limited disease and all were operated on by one surgeon (RL). Patients dying within the first week of surgery were excluded as were those in whom the cause of death was unknown.

**Table I**

| *Blood group* | *Number of patients* | *Observed deaths* | *Expected deaths* |
|---|---|---|---|
| A | 28 | 10 | 10.94 |
| B | 10 | 7 | 3.14 |
| AB | 5 | 4 | 1.09 |
| O | 43 | 11 | 16.82 |

Further analysis revealed no association between survival and either perioperative blood transfusion or rhesus status. Detailed analysis of the survival of the various histological types within each blood group was precluded by the small numbers involved.

The explanation for the poorer survival of patients whose blood group is B or AB is unclear, but these findings suggest that genetic factors may play a role in determining the prognosis of patients with lung cancer.

Yours etc.

T.E. Roberts[1], P. Hasleton[2],
R. Swindell[3] & R. Lawson[4]
[1]Department of Medicine,
University Hospital of South Manchester;
Departments of [2]Pathology & [4]Surgery,
Wythenshawe Hospital, Withington; and
[3]Department of Medical Statistics,
Christie Hospital & Holt Radium Institute,
Manchester, UK.

### References

AIRD, I., BENTALL, H.H. & ROBERTS, J.A.F. (1953). Relationship between cancer of the stomach and the ABO groups. *Br. Med. J.*, **i:** 799.

ASHLEY, D.J.B. (1969). Blood groups and lung cancer. *J. Med. Genet.*, **6,** 183.

CAMERON, J.M. (1958). Blood groups in tumours of salivary tissue. *Lancet*, **i:** 239.

JAKOUBKOVÁ, J. & MÁJSKY, A. (1965). Blood groups and neoplastic disease. *Neoplasia*, **12,** 611.

RACE, R.R. & SANGER, R. (1975). *Blood Groups in Man.* Blackwell Scientific: Oxford.